# Integrative genomics analysis identifies five promising genes implicated in insomnia risk based on multiple omics datasets

**DOI:** 10.1042/BSR20201084

**Published:** 2020-09-02

**Authors:** Haozhen Sun, Jianhua Zhang, Yunlong Ma, Jingjing Liu

**Affiliations:** 1 Department of Clinical Pharmacy, the First Affiliated Hospital, Zhejiang University School of Medicine, 79 Qingchun Road, Hangzhou 310003, P. R. China; 2 Department of psychological consulting, the logistics Service Center of Municipal Building, Hangzhou 310019, P. R. China; 3 Institute of Biomedical Big Data, Wenzhou Medical University, Wenzhou 325027, Zhejiang, China; 4 School of Biomedical Engineering, School of Ophthalmology and Optometry and Eye Hospital, Wenzhou Medical University, Wenzhou 325027, Zhejiang, China; 5 Department of Nursing, the First Affiliated Hospital, Zhejiang University School of Medicine, 79 Qingchun Road, Hangzhou 310003, P. R. China

**Keywords:** gene expression, genetic variants, GWAS, Insomnia, risk genes, sleep disease

## Abstract

In recent decades, many genome-wide association studies on insomnia have reported numerous genes harboring multiple risk variants. Nevertheless, the molecular functions of these risk variants conveying risk to insomnia are still ill-studied. In the present study, we integrated GWAS summary statistics (*N*=386,533) with two independent brain expression quantitative trait loci (eQTL) datasets (*N*=329) to determine whether expression-associated SNPs convey risk to insomnia. Furthermore, we applied numerous bioinformatics analyses to highlight promising genes associated with insomnia risk. By using *Sherlock* integrative analysis, we detected 449 significant insomnia-associated genes in the discovery stage. These identified genes were significantly overrepresented in six biological pathways including Huntington’s disease (*P*=5.58 × 10^−5^), Alzheimer’s disease (*P*=5.58 × 10^−5^), Parkinson’s disease (*P*=6.34 × 10^−5^), spliceosome (*P*=1.17 × 10^−4^), oxidative phosphorylation (*P*=1.09 × 10^−4^), and wnt signaling pathways (*P*=2.07 × 10^−4^). Further, five of these identified genes were replicated in an independent brain eQTL dataset. Through a PPI network analysis, we found that there existed highly functional interactions among these five identified genes. Three genes of *LDHA* (*P*=0.044), *DALRD3* (*P*=5.0 × 10^−5^), and *HEBP2* (*P*=0.032) showed significantly lower expression level in brain tissues of insomnic patients than that in controls. In addition, the expression levels of these five genes showed prominently dynamic changes across different time points between behavioral states of sleep and sleep deprivation in mice brain cortex. Together, the evidence of the present study strongly suggested that these five identified genes may represent candidate genes and contributed risk to the etiology of insomnia.

## Introduction

Insomnia is characterized by persistent dissatisfaction with sleep, which may play central roles in the etiology of physical and mental health [1–3], including suicide [2], depression [4], and post-traumatic stress disorder [5]. In the general population, it is estimated that the prevalence of insomnia is approximately 10–20% [6–8]. Previous twin studies have documented that insomnia and sleep characteristics are highly influenced by genetic factors [9–11]. The heritability rates were estimated to be 59% in females, and 38% in males [12]. Thus, growing studies show considerable interest in identifying the genetic basis of insomnia.

With the advance of technique, genome-wide association study (GWAS) is widely applied and considered as an effective method that could simultaneously examine the genetic association signals from millions of SNPs with complex traits of interest. In recent years, numerous genetic variants based on multiple GWAS [13–17], have been identified to be associated with insomnia complaints and insomnia symptoms. There were more than 200 genomic loci from these reported GWAS including a wealth of information that are capable of revealing new risk genes and biological pathways implicated in insomnia risk [13–15]. Nevertheless, these biological effects of significant genetic variants from GWAS studies on insomnia risk remain unclear. Meanwhile, in light of the stringent genome-wide threshold of statistical significance employed, there were a great number of genetic loci with small effect size difficult to be identified in a single GWAS study. Therefore, more relevant investigations on uncovering the biological mechanism of genetic variants with small-to-modest effect size may contribute to understanding the missing heritability of insomnia.

Although there are many insomnia-related genetic loci to be detected, accumulating studies with strong evidence have reported that abnormal expression in risk genes plays an important role in the pathogenesis of complex diseases [13,18–21], including insomnia. In addition, multiple studies [22–25] have recently applied systematically integrative methods to combine expression quantitative trait loci (eQTL) data and GWAS summary statistics for identifying the underlying regulatory effect of the reported risk SNPs from previous GWAS. A recent study reported by He and his co-workers [22] demonstrated a Bayesian statistical inference method called *Sherlock* to systematically uncover the cis- and trans-regulatory effects of susceptibility genes on complicate disorders based on GWAS summary statistics data and eQTL data. Based on this effective and powerful approach, a growing number of studies on different phenotypes, such as major depressive disorders [26,27], gout disease [28], and schizophrenia [29], have identified numerous new susceptibility genes, which cannot be detected in GWAS alone.

To the best of our knowledge, there is no systematically integrative study integrating the large-scale GWAS and eQTL data to reveal the insomnia-associated risk genes. Thus, the primary aim of the current investigation is to determine whether expression-associated SNPs could confer risk to insomnia and detect insomnia-associated risk genes by using the *Sherlock* approach based on both eQTL and GWAS data. Furthermore, we adopted quantities of bioinformatics tools based on multiple independent omics data to validate our findings.

## Methods

### Insomnia GWAS summary data

We applied a large-scale insomnia-related GWAS summary dataset from the UK Biobank database [13] for identifying susceptibility SNPs and genes. There is a total of 386,533 individuals with 109,402 cases and 277,131 controls of European descent included in the current investigation. Since the number of 40,000 samples allows for more than 90% high-power detection of the small effect sizes of genetic variants [30], we infer the present study has sufficient power for identifying risk genetic variants. In this chosen population, the insomnia prevalence was 28.3%. All included subjects signed informed consent. Both phenotypic information and DNA samples were collected from participants. The phenotype of insomnia was recorded according to the following question: “Do you have trouble falling asleep at night or do you wake up in the middle of the night?” The answers of the question were provided to subjects for choosing: “A: Never/rarely, B: Sometimes, C: Usually, D: Prefer not to answer. Genotype data were based on the combined UK10K and 1000 Genome Projects reference panel (hg19) for imputation. In total, there were a number of 10,862,567 genetic variants and related *P* values employed as input in current *Sherlock* Bayesian inference analysis.

### GWAS data based on Null phenotype

In order to avoid the influence of random events, we employed published GWAS data (*N*=3960) [31] to construct a fake insomnia-based GWAS data as a negative control. With regard to the constructed GWAS dataset, we randomly assigned the disease status (namely, insomnia or control) into each individual of 3960 samples by using the method of RANDBETWEEN (“insomnia”, “control”) in the Microsoft Excel. We called the randomly assigned insomnia as Null trait. The statistical analysis utilized the widely used tool, namely PLINK v1.07 [32], based on the logistic regression model. In light of the assumption that there were no true genetic effects of the GWAS on Null phenotype, the relatively small sample size is not an issue.

### Brain eQTL data

Considering that insomnia is the second most prevalent mental disorder that may be susceptible to the aberrant function of the brain, it is plausible to assume that brain tissue is the most suitable sample for integrative analysis of identifying insomnia-associated risk genes. Thus, we employed human brain prefrontal cortex tissue-based expression data from a previously reported study by Myers and co-workers [33] to perform an eQTL analysis. There were 193 neuropathologically normal brain samples without clinical history of psychiatric phenotypes or other neurologic traits included in subsequent analysis. All enrolled participants were European origins based on self-report. The expression profiles were obtained with the adoption of an Illumina HumanRefseq-8 Expression BeadChip, and the genome-wide genotype data were genotyped with the use of an Affymetrix Human Mapping 500K Array Set.

### The Bayesian statistical method of Sherlock analysis

Since the vast majority of disease-associated genetic variants identified by GWAS are mapped in non-coding genomic regions [34], it is reasonable to infer that these identified risk variants in non-coding regions are more likely to affect the expression level of a specific gene rather than the molecular function of its protein. Thus, we employed a *Sherlock* Bayesian-based inference analysis to integrate GWAS summary data on insomnia from Jansen and co-workers [13]. The procedures of *Sherlock* inference approach are described as following steps: first, the Sherlock tool will identify all SNPs that show significant association with gene expression (called as eSNP) from chosen eQTL data based on brain cortical samples reported by Myers and co-workers [33]. After the definition of eSNP, *Sherlock* will examine the association between eSNPs and insomnia with the use of GWAS summary data from Jansen et al. [13]. A positive score would be given to an eSNP if this eSNP is also significantly associated with insomnia based on GWAS data. A negative score would be recorded if this eSNP shows a non-significant association with insomnia. There would be no score to be recorded if the SNP only shows significant association with insomnia but no prominent signal for alterations in gene expression. The total score of a specific gene is based on the score of each eSNP by combining evidence from GWAS and eQTL data. The logarithm of the Bayes factor (LBF) for a specific gene is a crucial indicator to determine whether the gene contributes to insomnia risk. The LBF is computed with the use of *Sherlock* Bayesian-based analysis by integrating the evidence from GWAS summary statistics and eQTL. The larger value of LBF represents the higher probability that the gene can convey risk to insomnia. Considering that existing traditional analyses often ignore SNPs with moderate effect size, *Sherlock* Bayesian-based analysis is an effective approach to systematically integrate SNP with moderate-to-strong effect size from GWAS and eQTL data. Bonferroni correction method was employed to correct the *P* values.

### Pathway-based enrichment analysis

To explain the biological function of the prioritized insomnia-related risk genes from *Sherlock* Bayesian-based analysis, we utilized an easy use plug-in of Cytoscape platform [35] called ClueGO [36] to generate a functional organized pathway-term network. First, we performed a pathway-based analysis depending on a popular public source of the Kyoto Encyclopedia of Genes and Genomes (KEGG). By using over-representation analysis, we could identify and prioritize functional associations between chosen genes and biological pathways. Furthermore, we performed a Gene Ontology (GO) analysis including three categories of GO terms: molecular function, cellular component, and biological process. The method of “*GO Term Fusion”* was used to reduce the redundancies among GO terms. The hypergeometric test was used to calculate *P* value and Bonferroni step down correction was used for multiple testing.

### Phenotype- and drug-related gene set enrichment analysis

To explore whether identified genes were significantly enriched in gene sets related to phenotypes or drugs, we utilized the web-based tool of WebGestalt [37] to perform functional enrichment analysis based on the resources of GLAD4U [38], DrugBank [39], and the Human Phenotype Ontology [37]. The web-access tool has three main functions of over-representation analysis, network topology-based analysis, and gene set enrichment analysis. Here we used the function of overrepresentation analysis to analyze the submitted genes identified from the *Sherlock* integrative analysis. Current enrichment analysis utilized all genome protein-coding genes as background genes. We only selected the gene size of each gene set ranging from 5 to 2000 for the current analysis. The method of the Benjamini–Hochberg false discovery rate was employed for adjustment.

### Validation eQTL datasets using Sherlock integrative analysis

To further replicate the authenticity of these identified insomnia-associated genes, we re-performed the *Sherlock* integrative analysis with the use of an independent eQTL dataset (i.e. 136 brain cortex samples) from GTEx portal (data release v7) [40]. RNA-sequencing was used for quantifying the level of RNA expression, and the Illumina OMNI 5M SNP Array was used for SNP genotyping. Based on the additive genetic model, eQTL analysis was conducted through the tool of Matrix eQTL [41] using linear regression analysis. For *Sherlock* Bayesian-based analysis, all the parameters were set to be the same with those in discovery eQTL data. The *P* values calculated from *Sherlock* were corrected with the application of the Bonferroni correction.

### MAGMA gene-based analysis

Furthermore, we intended to perform a gene-based enrichment analysis of the above-used GWAS summary dataset on insomnia [13] by adopting an independent approach of the Multi-marker Analysis of GenoMic Annotation (MAGMA). The IDs and *P* values of all SNPs were utilized as submitted information for the MAGMA tool. For revealing the multi-variant convergent genetic effects, the multiple regression model was utilized to integrate the linkage disequilibrium among SNPs within a specific genomic region. The definition that an SNP belongs to a specific gene depended on the location of the SNP. Whether it’s mapped into the gene body or a region extended ±20 kb downstream or upstream of the gene [42]. More detailed illustrations of the MAGMA are demonstrated in the official website of https://ctg.cncr.nl/software/magma. The SNP–SNP linkage disequilibrium information was computed as reference for the 1000 Genome European Panel, and the location of each SNP in the present study was referred to as the Human Genome Build 37.

### PPI network-based analysis

Numerous studies have been published to show that susceptibility genes for complex diseases are predisposed to be collectively interacted [20,43,44]. Furthermore, network-based analytic approaches have been widely used to search for functional patterns of identified genes associated with traits of interest [45,46]. Therefore, we performed a protein–protein interaction (PPI) network-based analysis of these identified insomnia-associated risk genes by using the GeneMANIA software [47], which is a user-friendly tool for speculating the functions of inputted genes and prioritizing the promising genes for further molecular experiments. This tool could extend the identified genes with functionally similar genes by integrating available proteomics and genomics data.

### Replication of candidate gene expression in brain tissue of insomnia patients

Under the assumption that aberrant expression of genes may convey risk to complex diseases, *Sherlock* Bayesian-based analysis is used to identify disease-associated risk genes. To determine whether these five identified susceptibility genes’ expression show a significant difference between insomnia and control brain samples, we downloaded one available RNA expression dataset by using Affymetrix Human Genome U133+ 2.0 chip from the NCBI’s GEO database (accession number: GSE40562) and performed a differential gene expression (DGE) analysis. For this dataset [19], there were three insomnia patients enrolled in the presetn study, and total RNA of the thalamus and the parietal cortex of insomnia patients were extracted using RNeasy Mini Kit (Qiagen) according to the manufacturer’s protocol. The Ethical Committee of National Institute for Viral Disease Prevention and Control, China CDC, approved for those human brain samples used in investigation. More detailed information on these samples including their genetic, pathogenic, and neuropathological features was reported in previous studies [48,49]. The web-based tool of GEO2R [50] was used to calculate the expression difference between insomnia and control. *P*-value < 0.05 was considered to be significant. The co-expression patterns of five identified genes were analyzed by the Pearson correlation analysis, and the *Corrplot* R package was used for visualization. The R script used for this analysis is shown in the github website (https://github.com/mayunlong89/insomnia/blob/master/coexpression.r).

### Temporal changes in the expression of candidate genes in mice brain cortex

To explore whether these identified candidate genes have significantly temporal changes in expression patterns in brain cortex between sleep and wake (sleep deprivation), we performed a differential expression pattern analysis at different time points by downloading the RNA expression data from NCBI GEO database (accession number: GSE6514). For this dataset, the male mice (C57BL/6J) at age of 10 ± 1 week were used in the experiments. With a dark–light cycle of 12 h, chosen mice were housed in a pathogen-free, humidity- and temperature-controlled room. These male mice were subjected to 14 days of acclimatization for establishing a nighttime feeding pattern. The detailed experimental information is recorded in a previous article [51]. The sleep deprivation procedure was initiated at lights-on with gentle handling. Five sleep-deprived mice were killed at each time point of 3, 6, 9, and 12 h of total sleep deprivation. Similarly, five undisturbed sleeping mice were killed at the same diurnal time points (i.e. 3, 6, 9, and 12 h) as sleep-deprived mice. Additionally, at the time of lights-on at 7:00 AM called as anchor time, five control mice were killed. The Affymetrix GeneChip Mouse Genome 430 2.0 array with >45,000 probe sets and ∼34,000 well-annotated genes. The intensity data of probes were analyzed with the application of the affy package of the R software, which is used to evaluate the quality of expression and generate summary measures of expression. Student’s *t*-test was used to calculate the expression difference between sleep and wake at each time point.

## Results

### Sherlock Bayesian-based analysis prioritizes insomnia-associated risk genes

**Figure 1:**
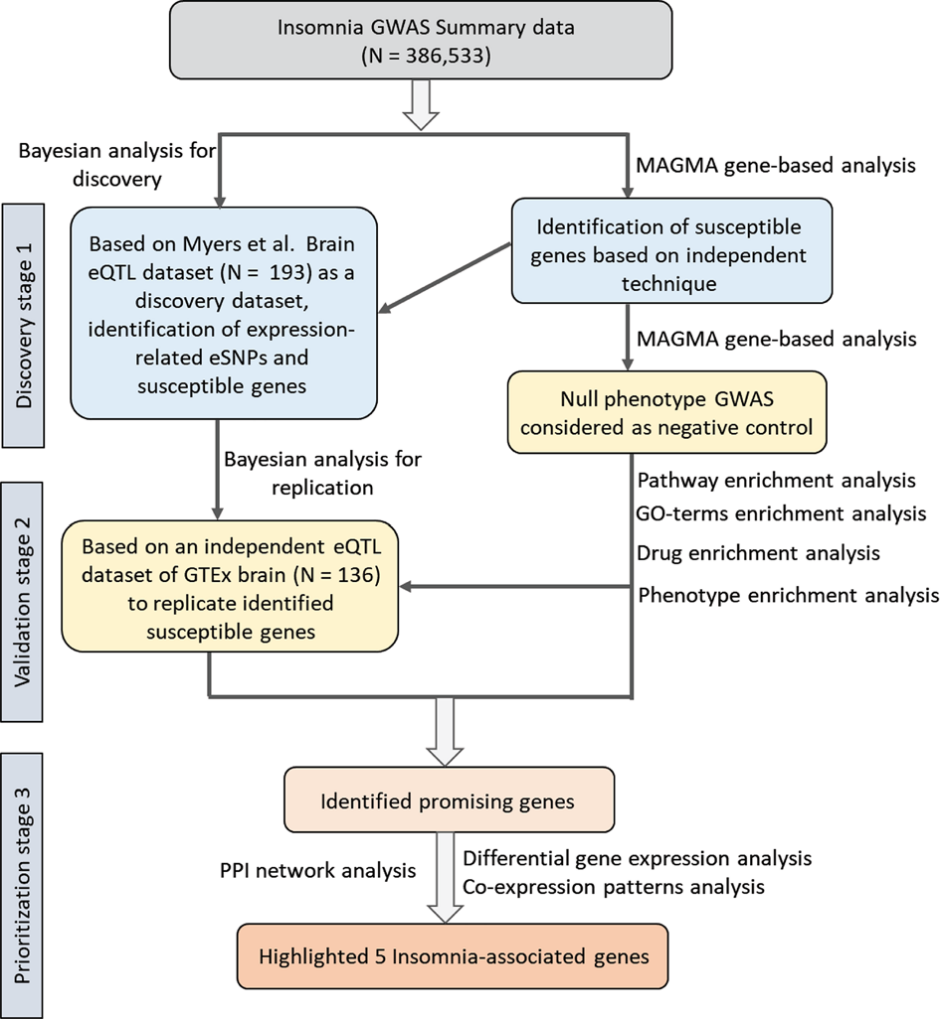
The workflow of identifying and prioritizing the insomnia risk genes

The workflow of our current study is shown in Figure 1. First, we employed *Sherlock* analysis to explore the association between SNP and expression by integrating insomnia-related GWAS summary statistics based on 386,533 samples and brain eQTL data based on 193 samples. Based on this Bayesian method of *Sherlock*, a total of 449 genes were found to be nominally significantly associated with insomnia risk by alteration in its expression (*P*-value < 0.05, Table 1, and Supplementary Table S1). For example, the top-ranked genes of *FOXF2* (simulated *P*=8.89 × 10^−6^), *FAM193A* (simulated *P*=1.22 × 10^−4^), *PAIP1* (simulated *P*=3.33 × 10^−4^), *MPG* (simulated *P*=3.67×10^−4^), *INO80* (simulated *P* = 5.00 × 10^−4^), *VPS13B* (simulated *P* = 8.89 × 10^−6^), and *TGFB3* (simulated *P* = 8.89 × 10^−6^) with supportive eSNPs conveying risk to insomnia (Table 1). Among them, 20 genes that are associated with insomnia or sleep-related phenotypes have been documented in the GWAS catalog database (Supplementary Table S1). For example, Spada and co-workers reported the rs62388641 in the *FOXF2* gene (*P*=1.0 × 10^−6^) is suggestively associated with daytime sleep phenotypes [52].

**Table 1:** Top-ranked 20 insomnia-associated genes identified by *Sherlock* integrative analysis

Gene symbol	Supporting SNP^1^	eSNP-based *P* value^2^	GWAS-based *P* value^3^	*Sherlock*-based *P* value^4^
*FOXF2*	rs4836269	9.96 × 10^−6^	0.067	8.89 × 10^−6^
*FAM193A*	rs7109421	9.92 × 10^−6^	0.010	1.22 × 10^−4^
*PAIP1*	rs11173336	9.87 × 10^−6^	0.018	3.33 × 10^−4^
*MPG*	rs1867116	9.96 × 10^−6^	0.076	3.67 × 10^−4^
*INO80*	rs4477668	9.72 × 10^−6^	0.49	5.00 × 10^−4^
*VPS13B*	rs6017342	9.74 × 10^−6^	0.61	5.98 × 10^−4^
*TGFB3*	rs2372321	9.99 × 10^−6^	0.029	7.78 ×10^−4^
*LOC283537*	rs9908305	9.83 × 10^−6^	0.076	8.00 × 10^−4^
*LOC155340*	rs810517	9.54 ×10^−6^	0.29	9.16 × 10^−4^
*GLUL*	rs1866877	9.70 × 10^−6^	0.034	1.05× 10^−3^
*RPS17*	rs7810180	9.65 × 10^−6^	0.0028	1.22 × 10^−3^
*ZNF621*	rs11135930	9.96× 10^−6^	0.040	1.33 × 10^−3^
*SMYD5*	rs951821	9.80 × 10^−6^	0.056	1.34 × 10^−3^
*PLLP*	rs418682	9.55 × 10^−6^	0.82	1.46 × 10^−3^
*RWDD2B*	rs8098365	9.67 × 10^−6^	0.57	1.60 ×10^−3^
*CAT*	rs2172962	9.42 ×10^−6^	0.59	1.74 × 10^−3^
*SMEK1*	rs286451	9.80 × 10^−6^	0.97	1.89 × 10^−3^
*NDUFS6*	rs1218887	9.36 × 10^−6^	0.16	1.90 ×10^−3^
*LYL1*	rs4694022	9.28 × 10^−6^	4.19×10^−6^	2.00 × 10^−3^
*FAF1*	rs4775953	9.30 × 10^−6^	0.0023	2.11 × 10^−3^

^1^SNP influences the expression level of risk gene.

^2^*P*-value from expression quantitative trait analysis of Myers et al.

^3^*P*-value from GWAS on insomnia of Jansen et al.

^4^*P*-value from calculation based on the *Sherlock* Bayesian integrative analysis. In light of *Sherlock* tool uses finite times of permutation test to calculate the *P* value for each gene, some of these top-ranked genes have distinct logarithm of the Bayes factor values but obtain the same rankings (namely, their *P* values are same).

### Identification of significantly enriched pathways

**Figure 2:**
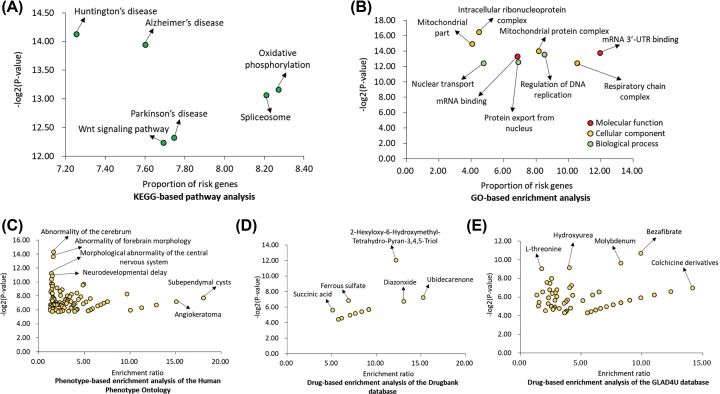
Functional enrichment analysis of insomnia-associated genes identified by Sherlock Bayesian analysis (**A**) Pathway enrichment analysis based on the KEGG database. (**B**) GO-terms enrichment analysis based on three wide-used terms of molecular function, cellular component, and biological process. (**C**) Phenotype-based enrichment analysis of the Human Phenotype Ontology based on the WebGestalt tool. (**D**) Drug-based enrichment analysis of the Drugbank database based on the WebGestalt. (**E**) Drug-based enrichment analysis of the GLAD4U database based on the WebGestalt.

Subsequently, we conducted a pathway analysis based on the KEGG source for these identified 449 insomnia-associated genes. There were six biological pathways significantly enriched by these inputted genes (Figure 2A and Supplementary Table S2; Corrected *P*-value < 0.05). Interestingly, these overrepresented pathways have been well-documented to be implicated in psychiatric disorders or neurodegenerative diseases [53–59]. For example, the pathways of Huntington’s disease (*P*=5.58 × 10^−5^), Alzheimer’s disease (*P*=5.58 × 10^−5^), Parkinson’s disease (*P*=6.34 × 10^−5^), spliceosome (*P*=1.17 × 10^−4^), oxidative phosphorylation (*P*=1.09 × 10^−4^), and wnt signaling pathway (*P*=2.07 × 10^−4^). Furthermore, we carried out a GO enrichment analysis according to three categories of GO terms. With respect to the category of molecular function (Figure 2B and Supplementary Table S3), we found that these identified insomnia-associated genes were significantly enriched in mRNA 3’-UTR binding (*P*=7.39 × 10^−5^) and mRNA binding (*P*=1.01 × 10^−4^). With regard to the category of cellular component (Figure 2B), five terms were significantly enriched; for example, mitochondrial part (*P*=3.22 × 10^−5^), mitochondrial protein complex (*P*=6.08 × 10^−5^), and mitochondrial membrane part (*P*=1.01 × 10^−4^). For the category of biological process (Figure 2B), we observed three significantly enriched terms: regulation of DNA replication (*P*=8.28 × 10^−5^), protein export from nucleus (*P*=1.65 × 10^−4^), and nuclear transport (*P*=1.82 × 10^−4^).

In addition, we utilized the WebGestalt software to conduct phenotype- and drug-based enrichment analysis. Interestingly, with regard to phenotype-focused enrichment analysis, we found a number of enriched gene sets related to several defined phenotypes (Figure 2C and Supplementary Table S4). For example, the gene sets were related to abnormality of the cerebrum (*P*=4.88 × 10^−5^), abnormality of forebrain morphology (*P*=7.66 × 10^−5^), morphological abnormality of the central nervous system (*P*=4.17 × 10^−4^), and neurodevelopmental delay (*P*=5.19 × 10^−4^). For drug-focused enrichment analysis, 83 gene sets related to drugs were significantly enriched based on two widely-used databases of DrugBank (Figure 2D and Supplementary Table S5) and GLAD4U (Figure 2E and Supplementary Table S6).

### Replication of identified risk genes using an independent eQTL dataset

**Figure 3:**
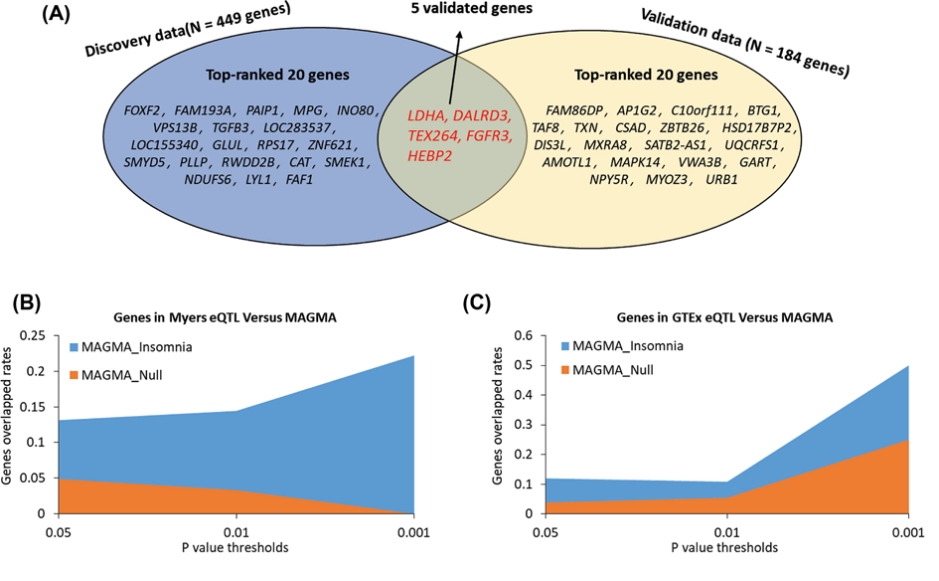
Identification of insomnia risk genes based on two independent eQTL datasets (**A**) Venn plot demonstrated the overlapped genes between discovery (Myers eQTL) and replication stage (GTEx eQTL). (**B**) Sherlock-identified genes from Myers eQTL data (the discovery stage) were obviously higher overlapped with genes identified from MAGMA analysis of GWAS on insomnia than those from MAGMA analysis of GWAS on null phenotype. (**C**) Sherlock-identified genes from GTEx eQTL data (the replication stage) were obviously higher overlapped with genes identified from MAGMA analysis of GWAS on insomnia than those identified from MAGMA analysis of GWAS on null phenotype.

For validation of above identified genes, we then reconducted the *Sherlock* Bayesian-based integrative analysis with the same parameter settings using independent brain cortex eQTL data (*N*=136). *Sherlock* Bayesian-based analysis identified 184 significant insomnia-associated genes (Supplementary Table S7). There were two genes of *C6orf201* and *AK5* reported to be associated with sleep-related traits [52,60]. By compared with genes identified from the discovery stage, we found that five significant genes were overlapped between discovery and replication stage; namely, *HEBP2* (simulated *P*=0.015), *LDHA* (simulated *P*=0.018), *TEX264* (simulated *P*=0.02), *FGFR3* (simulated *P*=0.023), and *DALRD3* (simulated *P*=0.029) (Figure 3A and Supplementary Table S8).

To further ensure the reliability of current investigation, we conducted MAGMA gene analyses for GWAS on insomnia and GWAS on Null phenotype (referred to as negative control). For comparing the findings between real and fake data, we employed three distinct thresholds of *P* values: 0.05, 0.01, and 0.001. At each threshold, we observed that *Sherlock*-identified genes (Myers eQTL and GTEx eQTL) were obviously higher overlapped with MAGMA-identified genes from GWAS on insomnia than those from GWAS on Null phenotype (Figure 3B,C).

### PPI network-based analysis of five insomnia-risk genes

**Figure 4:**
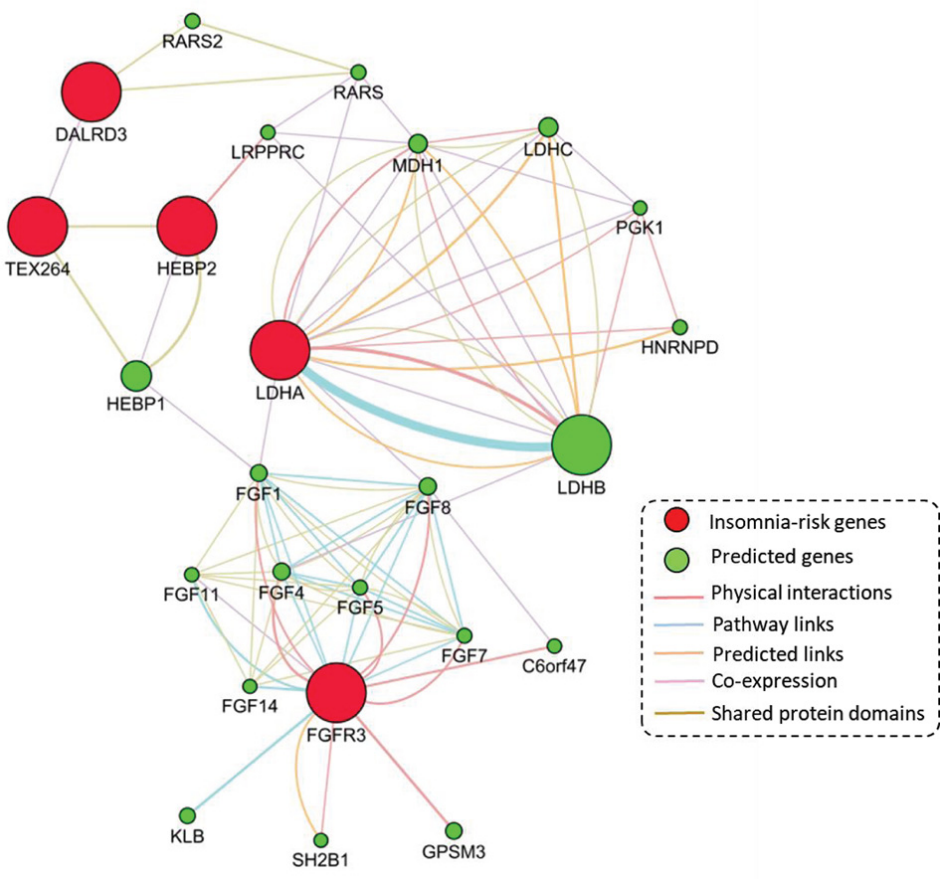
Protein–protein interactions network constructed by using five identified insomnia risk genes The five insomnia-associated risk genes were provided as query (red nodes) and a number of additional genes were predicted to be linked (green nodes). The interactions evidence was based on psychical interactions, pathway links, predicted links, co-expression, and shared protein domains.

To determine whether identified insomnia-risk genes functionally interacted together, we performed a PPI network-based analysis with the application of interactions of physical interactions, co-expression, predictions, pathways, and shared protein domains based on the well-documented database of GeneMANIA [47]. Figure 4 demonstrated that these identified insomnia-risk genes is generated a biological network, suggesting that there exist highly functional links among these identified risk genes. For example, two hub genes of *LDHA* and *FGFR3* have the most interactions with other genes (Figure 4). Additionally, the gene of *TEX264* shows evidence of shared protein domains with the insomnia-risk gene of *HEBP2*, and has co-expression evidence with *DALRD3* (Figure 4).

### Differential expression of identified risk genes between insomnia and control brain samples

**Figure 5:**
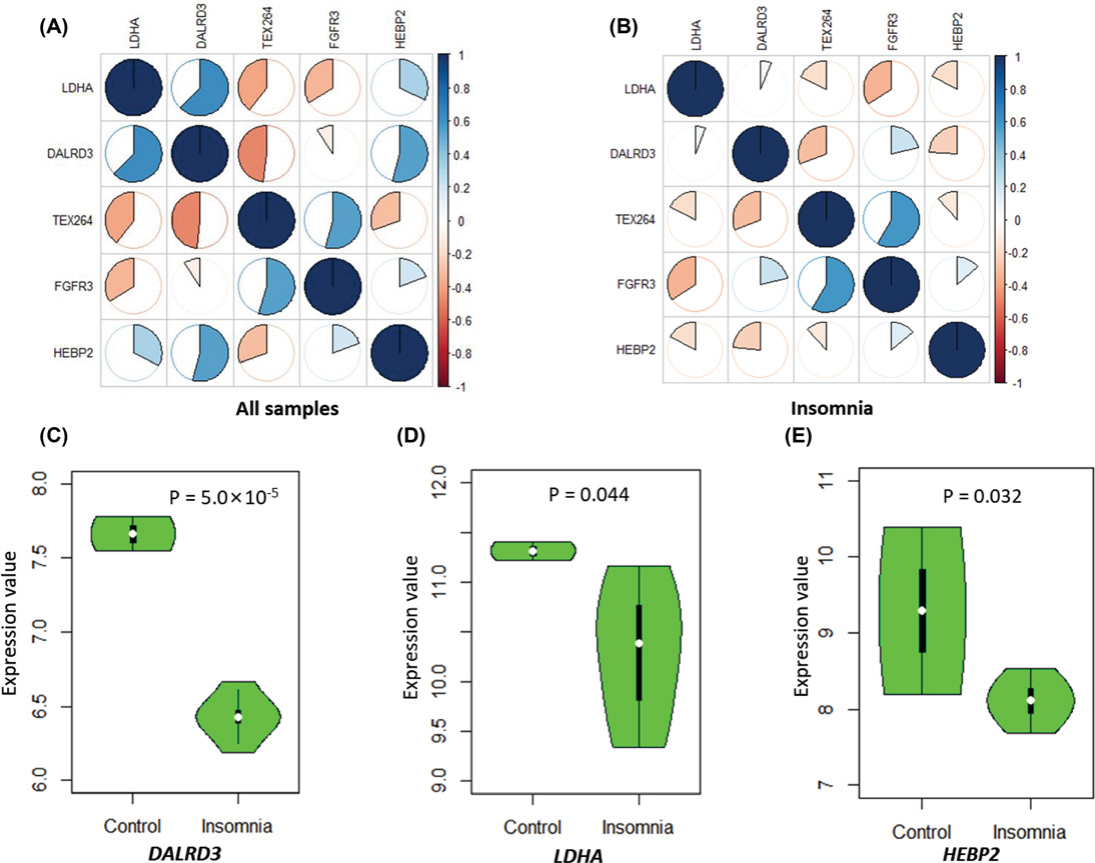
The differential expression patterns in the brain between insomnic patients and controls (**A**) Co-expression patterns of five susceptible genes in all samples based on the Pearson correlation analysis. (**B**) Co-expression patterns of five susceptible genes in insomnic patients based on the Pearson correlation analysis. (**C** and **D**) Boxplots show differential expression signatures of identified genes in the brain between insomnic patients and controls. (C) for *DALRD3*, (D) for *LDHA*, and (**E**) for *HEBP2.*

To determine whether the co-expression patterns among five identified genes were altered by disease status, we carried out a Pearson correlation analysis with the use of *corrplot* package for a visualization based on the RNA expression data from GSE40562 from the NCBI GEO database. Interestingly, we found prominent alterations of the co-expression relationships among five genes categorized by insomnia status (Figure 5A,B and Supplementary Tables S9–S10). For example, the positive correlation coefficient of *LDHA* with *DALRD3* was 0.62 in all samples, but it was largely reduced to 0.06 in insomnic patients. The negative correlation score of *LDHA* with *TEX264* was reduced from −0.40 in all samples to −0.18 in insomnic patients. Similarly, the negative correlation score between *DALRD3* and *TEX264* was decreased from −0.48 in all samples to −0.31 in insomnic patients. In addition, the co-expression correlation between *DALRD3* and *HEBP2* fundamentally changed from 0.54 in all samples to −0.24 in insomnic patients.

**Figure 6:**
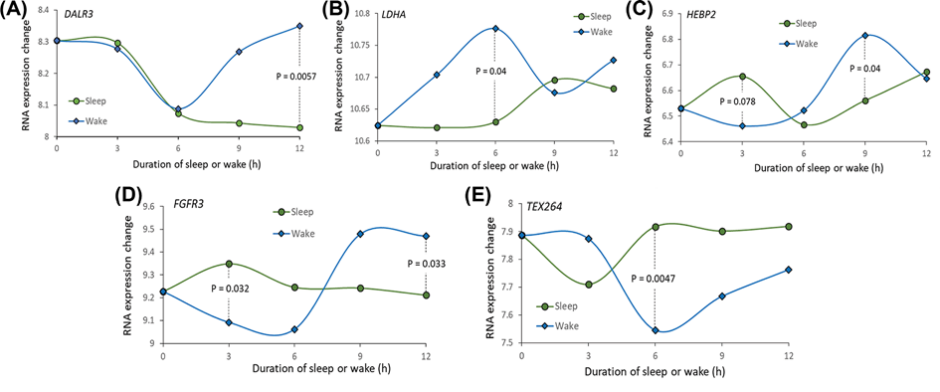
The temporal changes in the expression of five identified risk genes in the brain between behavioral states of sleep and sleep deprivation (**A**) for *DALRD3*, (**B**) for *LDHA*, (**C**) for *HEBP2*, (**D**) for *FGFR3*, (**E**) for *TEX264*.

Furthermore, by conducting a DGE analysis, we found that three genes of *DALRD3* (*P*=5.0 × 10^−5^), *LDHA* (*P*=0.044), and *HEBP2* (*P*=0.032) showed significantly down-regulated expression in insomnia brain samples compared with controls (Figure 5C–E and Supplementary Table S8). Unfortunately, there was no significant evidence for both *FGFR3* and *TEX264* between insomnia patients and controls (Supplementary Table S8). In addition, based on the dataset of GSE6514, we found that the expression patterns of these five genes between sleep and sleep deprivation remarkably changed across different time points in mice brain cortex (Figure 6A–E). For example, the gene of *DALR3* has similar decreased expression patterns of sleep and wake states before the time point of 6 h, but subsequently the gene expression is prominently increased in wake state and still decreased in sleep state (Figure 6A).

## Discussion

Insomnia disorder is one of the most prevalent mental disorders worldwide [61]. Multiple lines of evidence from family and twin studies have been reported to suggest the involvement of genetic components in the pathogenesis of insomnia [9–11]. To date, a few insomnia-related linkages and candidate genetic association studies with relatively small sample sizes have been carried out. With the development of high-throughput genotyping technologies and the increase in sample size, numerous highly significant genetic variants among more than 200 genomic loci have been identified to be associated with insomnia by using the GWAS approach [13–17]. However, it remains equivocal how these identified genetic variants convey susceptibility to insomnia. In view of suffering the influence of linkage disequilibrium, the vast majority of reported susceptibility loci contain many highly linked genetic variants with similar significant association signals, enhancing the difficulty to find the authentic causal variants. As we all know, most of the GWAS-identified genetic variants are mapped into non-coding regions of the genome [62,63]. Thus, there is a tough conundrum from GWAS left which is how a genetic variant change in the non-coding region could contribute to increased risk to a specific phenotype, such as insomnia. One possible explanation is that these identified genetic variants in non-coding regions are more likely to result in alterations of gene expression levels rather than in alterations of protein functions [34,64].

To address this issue, many researchers have developed various bioinformatics statistical approaches for data integration of the genetic variants information from GWAS summary statistics and eQTL datasets [22,64,65]. In the present study, we performed a *Sherlock* Bayesian-based integrative analysis of two independent brain eQTL datasets (*N*=329) and GWAS summary data on insomnia (*N*=386,533) to identify candidate gene conferring susceptibility to insomnia. The approach of GWAS scanning tens of millions of genetic variants is extensively applied to identify genomic regions harboring common SNPs that are significantly associated with traits of interest, including insomnia. Nevertheless, the burden of correction for multiple-testing from millions of SNPs remarkably limits the power of GWAS to confirm the associated risk SNPs. Many SNPs with small-to-modest effects that do not reach a genome-wide significance but is still pivotal to insomnia may be ignored by GWAS. In addition, GWAS alone could not infer whether these identified disease-associated SNPs have functional consequences. Thus, *Sherlock* Bayesian analysis used in the current investigation is an effective approach for identifying novel risk genes based on genetic and expression information, and has been widely employed in numerous diseases [22,26–29].

In the discovery stage, we observed 499 genes whose alterations in expression may be implicated in the etiology of insomnia by using *Sherlock* analysis. Numerous identified genes have been demonstrated to be associated with sleep-related traits or insomnia in previous studies. For example, the genes of *FOXF2* [52], *PLLP* [66], and *WWC1* [52]. More interestingly, these identified genes were over-represented in six biological pathways, which have been widely implicated in the etiology of neuropsychiatric or neurological disorders [53–59,67], including insomnia-related disease [68,69]. Of these pathways, three pathways of Huntington’s disease, Alzheimer’s disease, and Parkinson’s disease were derived from neurodegenerative disorders, which is the umbrella term for the progressive loss of structure or function of neurons. For example, Huntington’s disease is a neurodegenerative genetic disorder that leads to mental decline and behavioral symptoms [70]. Also, the other three significantly enriched pathways of spliceosome, oxidative phosphorylation, and wnt signaling pathway have been extensively reported to be involved in neurodegenerative disorders, such as autism [71–73], Alzheimer’s disease [74,75], Parkinson’s disease [76–78], and Huntington’s disease [79,80]. Consistently, previous studies [60] have reported that there existed shared genetics between sleep disturbance traits and neuropsychiatric disorders. Consistently, our phenotype-focused enrichment analysis also found these *Sherlock*-identified genes were significantly enriched in several phenotypes relevant to neurodevelopment or brain morphological abnormality, including abnormality of the cerebrum, abnormality of forebrain morphology, and morphological abnormality of the central nervous system. Thus, our results provided supportive evidence that insomnia has an impact on the quality of life in patients and may involve in the pathogenesis of various neurodegenerative disorders.

To validate and prioritize these identified genes, we reconducted the *Sherlock* Bayesian analysis with the use of an independent brain eQTL dataset. There were five promising genes of *DALRD3, LDHA, HEBP2, TEX264*, and *FGFR3* replicated. Meanwhile, we detected that the *Sherlock*-identified genes from both discovery and replication stage were prominently higher overlapped with MAGMA-discovered genes from GWAS on insomnia than that from GWAS on Null phenotype, indicating that these *Sherlock*-identified genes associated insomnia risk are probably due to genetic components rather than random chances. Through conducting the PPI network analysis, we noticed that these five genes collectively interacted with each other. The insomnia-associated gene of *HEBP2* has shared protein domains with the insomnia-associated gene of *TEX264* [81], and *TEX264* show evidence of co-expression with *DALRD3* [82]. The insomnia-associated gene of *LDHA* has co-expression links with predicted genes of *PGK1* and *RARS* [83]. By using co-expression analysis, we found that the gene–gene co-expression patterns were significantly different between insomnic patients and controls. Furthermore, we observed *DALRD3, LDHA*, and *HEBP2* are significantly lower expressed in insomnic patients than those in controls. Consistently, these five identified genes showed differential expression patterns between sleeping duration and sleep deprivation at different time points. Together, these findings provided consistent evidence to support that these newly identified genes have roles in the etiology of insomnia risk and may represent therapeutic targets for treating insomnia.

In the current investigation, by using numerous bioinformatics analyses including *Sherlock* Bayesian analysis, MAGMA gene enrichment analysis, pathway enrichment analysis, drug-based enrichment analysis, PPI network-based analysis, co-expression analysis, DGE-based analysis, and differential time point-based expression analysis based on multilayer data from various sources, we identified five novel susceptible genes associated with insomnia risk. With regard to five identified genes, there existed a bulk of evidence to demonstrate their molecular functions. The protein, encoded by *LDHA* gene, catalyzes the conversion of L-lactate and NAD to pyruvate and NADH in the final step of anaerobic glycolysis. The *LDHA* gene has been reported to be implicated in various neurodegenerative disorders [84,85]. For example, Newington and co-workers [84] have shown that the overexpression of *LDHA* in a rat B12 cell line conveys resistance to amyloid β and other neurotoxins, which may elucidate why some people tolerate high levels of amyloid β deposition without the development of Alzheimer’s disease. Furthermore, a single conserved exon 5 haplotype in *LDHA* is remarkably associated with the risk of panic disorder, which is a type of anxiety disorder [86]. The mRNA expression levels of *LDHA* gene increased in major depressive disorder patients in both depressive state and remissive state in comparison with healthy control subjects [87]. For the gene of *DALRD3*, it encodes a protein with a DALR anticodon binding domain similar to that of class la aminoacyl tRNA synthetases. The abnormal expression of *DALRD3* has been reported to be significantly associated with sleep-related phenotypes (*P*=0.033) [88]. The *FGFR3* gene, encoding a member of the fibroblast growth factor receptor family, has been reported to be exclusively expressed in the locus coeruleus in patients with major depressive disorder [89]. Previous studies have demonstrated that skeletal dysplasia patients with Asn540Lys mutation in the *FGFR3* gene have been documented to accompany with medial temporal lobe dysgenesis and epilepsy [90–92].

In conclusion, the current comprehensive study provides multiple lines of evidence for supporting *DALRD3, LDHA, HEBP2, TEX264*, and *FGFR3* as insomnia-associated genes whose abnormal expression level may convey risk to insomnia. Our findings indicate that individuals suffering from insomnia may be more vulnerable to various neurodegenerative disorders. Furthermore, we link insomnia risk variants to susceptible genes and biological pathways, offering a possible explanation of biological mechanism between genetic variation and insomnia risk. Further molecular experiments are warranted to investigate the molecular functions of identified genes and risk variants.

## Supplementary Material

Supplementary Tables S1-S10

## Abbreviations

DGE: differential gene expression
eQTL: expression quantitative trait loci
LBF: logarithm of the Bayes factor
MAGMA: Multi-marker Analysis of GenoMic Annotation
PPI: protein–protein interaction
