# Supplementary material for: Integrative genomics analysis identifies five promising genes implicated in insomnia risk based on multiple omics datasets
Source: Biosci Rep. 2020 Sep 2;40(9):BSR20201084. doi: 10.1042/BSR20201084 (PMC7468094; doi:10.1042/BSR20201084)
Supplement: Supplementary Tables S1-S10 [file BSR-2020-1084_supp.pdf]

Supplemental Tables

Supplemental Table S1. ***Sherlock*** **integrative analysis identifies 449 genes as insomnia risk genes in discovery samples**

**Gene**	**LBF**	***Sherlock*-based**	**GWAS Catalog**
FOXF2	-0.046	8.89E-06	Reported gene
FAM193A	-0.047	1.22E-04	Novel gene
PAIP1	-0.047	3.33E-04	Novel gene
MPG	-0.048	3.67E-04	Novel gene
INO80	-0.048	5.00E-04	Novel gene
VPS13B	-0.048	5.98E-04	Novel gene
TGFB3	-0.048	7.78E-04	Novel gene
LOC283537	-0.048	8.00E-04	Novel gene
LOC155340	-0.048	9.16E-04	Novel gene
GLUL	-0.048	1.05E-03	Novel gene
RPS17	-0.049	1.22E-03	Novel gene
ZNF621	-0.049	1.33E-03	Novel gene
SMYD5	-0.049	1.34E-03	Novel gene
PLLP	-0.049	1.46E-03	Reported gene
RWDD2B	-0.049	1.60E-03	Novel gene
CAT	-0.049	1.74E-03	Novel gene
SMEK1	-0.049	1.89E-03	Reported gene
NDUFS6	-0.049	1.90E-03	Novel gene
LYL1	-0.049	2.00E-03	Novel gene
FAF1	-0.049	2.11E-03	Novel gene
TNFSF13	-0.049	2.29E-03	Novel gene
HNRNPC	-0.050	2.35E-03	Novel gene
GRSF1	-0.050	2.52E-03	Novel gene
BCAS1	-0.050	2.58E-03	Novel gene
WWC1	-0.050	2.73E-03	Reported gene
VPS52	-0.050	2.85E-03	Novel gene
SLC26A11	-0.050	3.00E-03	Novel gene
PLCB1	-0.050	3.11E-03	Reported gene
C15orf41	-0.050	3.19E-03	Novel gene
PCBD1	-0.050	0.003	Novel gene
RPS21	-0.050	0.003	Novel gene
CCT8	-0.050	0.003	Novel gene
KPTN	-0.050	0.004	Novel gene
SPOCK2	-0.050	0.004	Novel gene
SLC35A1	-0.051	0.004	Novel gene
CDC27	-0.051	0.004	Novel gene
DAPK1	-0.051	0.004	Reported gene
AMT	-0.051	0.004	Reported gene
MYO5C	-0.051	0.004	Novel gene
PDE4C	-0.051	0.004	Novel gene
FBXO28	-0.051	0.005	Novel gene
HPCL2	-0.051	0.005	Novel gene
GRAMD1A	-0.052	0.005	Novel gene
CYB561D1	-0.052	0.005	Novel gene
S1PR5	-0.052	0.005	Novel gene
IL18	-0.052	0.005	Novel gene

ZCRB1	-0.052	0.005	Novel gene
NUBPL	-0.052	0.005	Novel gene
NASP	-0.052	0.005	Novel gene
LOC400810	-0.052	0.006	Novel gene
B3GAT1	-0.052	0.006	Novel gene
PIK3IP1	-0.052	0.006	Novel gene
GRAMD3	-0.052	0.006	Novel gene
PIEZO2	-0.053	0.006	Novel gene
PSEN1	-0.053	0.006	Novel gene
TNS3	-0.053	0.006	Novel gene
RBBP7	-0.053	0.006	Novel gene
LOC170371	-0.053	0.006	Novel gene
LIPE	-0.053	0.007	Novel gene
TRAPPC10	-0.053	0.007	Novel gene
C7orf63	-0.053	0.007	Novel gene
CHD8	-0.053	0.007	Novel gene
MFSD5	-0.053	0.007	Novel gene
BRODL	-0.053	0.007	Novel gene
C14orf43	-0.053	0.007	Novel gene
CCDC106	-0.053	0.007	Novel gene
FA2H	-0.053	0.007	Novel gene
LOC143425	-0.053	0.008	Novel gene
C19orf10	-0.054	0.008	Novel gene
TNFAIP2	-0.054	0.008	Novel gene
LOC285148	-0.054	0.008	Novel gene
MFF	-0.054	0.008	Novel gene
SLC25A38	-0.054	0.008	Novel gene
KCNMA1	-0.054	0.008	Novel gene
ANKRD37	-0.054	0.008	Novel gene
LOC348094	-0.054	0.008	Novel gene
CARHSP1	-0.054	0.009	Novel gene
RAB33A	-0.054	0.009	Novel gene
SEZ6L2	-0.054	0.009	Novel gene
NPLOC4	-0.054	0.009	Novel gene
SNX27	-0.055	0.009	Novel gene
LDHA	-0.055	0.009	Novel gene
PFN2	-0.055	0.009	Novel gene
TMEM209	-0.055	0.009	Novel gene
ZNF668	-0.055	0.009	Novel gene
SMARCA4	-0.056	0.010	Novel gene
TST	-0.056	0.010	Novel gene
GPHN	-0.056	0.010	Novel gene
LOC399917	-0.056	0.010	Novel gene
ZADH2	-0.056	0.010	Novel gene
DARC	-0.056	0.010	Novel gene
SESN1	-0.056	0.010	Novel gene
SAMD4B	-0.056	0.010	Novel gene
BCORL1	-0.056	0.010	Novel gene
DENND1B	-0.056	0.011	Novel gene
EFCAB6	-0.056	0.011	Novel gene
LOC284347	-0.056	0.011	Novel gene
FRMD6	-0.056	0.011	Novel gene
VSIG1	-0.057	0.011	Novel gene

C19orf53	-0.057	0.011	Novel gene
ATP5G1	-0.057	0.011	Novel gene
DALRD3	-0.057	0.011	Novel gene
ZNF518B	-0.057	0.011	Novel gene
SPP1	-0.057	0.012	Novel gene
PTPLB	-0.057	0.012	Novel gene
LOC401155	-0.057	0.012	Novel gene
TAF4	-0.057	0.012	Novel gene
EMX2	-0.057	0.012	Novel gene
LOC401260	-0.057	0.012	Novel gene
C4orf29	-0.058	0.012	Novel gene
EIF3FP2	-0.058	0.012	Novel gene
ZNF22	-0.058	0.012	Novel gene
HSPA12A	-0.058	0.013	Novel gene
CYB5R2	-0.058	0.013	Novel gene
DLX6-AS1	-0.058	0.013	Novel gene
NMT2	-0.058	0.013	Novel gene
STS	-0.058	0.013	Novel gene
LOC401528	-0.058	0.013	Novel gene
FKBP8	-0.059	0.013	Novel gene
NFXL1	-0.059	0.013	Novel gene
CLDN5	-0.059	0.013	Novel gene
SEC62	-0.059	0.014	Novel gene
ABL1	-0.059	0.014	Novel gene
LZIC	-0.059	0.014	Novel gene
RAB40C	-0.059	0.014	Novel gene
SIPA1L2	-0.059	0.014	Novel gene
PRSS23	-0.060	0.014	Novel gene
RPAP1	-0.060	0.014	Novel gene
RHBDD2	-0.060	0.014	Novel gene
PTPN4	-0.060	0.014	Novel gene
COX4I1	-0.060	0.015	Novel gene
PIEZO1	-0.060	0.015	Novel gene
FABP6	-0.060	0.015	Novel gene
SDCBP2	-0.060	0.015	Novel gene
THY1	-0.060	0.015	Novel gene
SGK1	-0.060	0.015	Novel gene
ZNF554	-0.060	0.015	Novel gene
SLC20A2	-0.060	0.015	Novel gene
CNRIP1	-0.060	0.015	Novel gene
FUCA1	-0.060	0.016	Novel gene
HNRNPR	-0.060	0.016	Novel gene
GTPBP4	-0.060	0.016	Novel gene
SNX11	-0.061	0.016	Novel gene
BRPF3	-0.061	0.016	Novel gene
NDST2	-0.061	0.016	Novel gene
CHCHD3	-0.061	0.016	Reported gene
SLC4A8	-0.061	0.016	Novel gene
CDK18	-0.061	0.016	Novel gene
CPNE8	-0.061	0.017	Novel gene
LOC342808	-0.061	0.017	Novel gene
KRTCAP2	-0.061	0.017	Novel gene
IFIH1	-0.061	0.017	Novel gene

TUBG1	-0.061	0.017	Novel gene
VAC14	-0.061	0.017	Novel gene
POPDC3	-0.062	0.017	Novel gene
TXN2	-0.062	0.017	Novel gene
EFEMP1	-0.062	0.017	Novel gene
SSR4	-0.062	0.018	Novel gene
NOC3L	-0.062	0.018	Novel gene
ALDH9A1	-0.062	0.018	Novel gene
NUP107	-0.062	0.018	Novel gene
FOXQ1	-0.062	0.018	Reported gene
ECSIT	-0.062	0.018	Novel gene
FXR2	-0.062	0.018	Novel gene
ITGAX	-0.062	0.018	Novel gene
LAPTM5	-0.062	0.018	Novel gene
ZBED1	-0.062	0.018	Novel gene
XPO5	-0.062	0.019	Novel gene
PCBP1	-0.062	0.019	Novel gene
LOC222901	-0.062	0.019	Novel gene
SERTAD2	-0.063	0.019	Novel gene
NDUFS5	-0.063	0.019	Novel gene
PPIL4	-0.063	0.019	Novel gene
C9orf9	-0.063	0.019	Novel gene
RAPGEF2	-0.063	0.019	Novel gene
DONSON	-0.063	0.020	Novel gene
RGS14	-0.063	0.020	Novel gene
SMG5	-0.063	0.020	Novel gene
LOC91526	-0.063	0.020	Novel gene
PLCB2	-0.063	0.020	Novel gene
RIOK1	-0.063	0.020	Novel gene
TXNRD2	-0.063	0.020	Novel gene
LOC51248	-0.063	0.020	Novel gene
TDG	-0.063	0.020	Novel gene
PPARD	-0.063	0.021	Novel gene
ARMCX3	-0.063	0.021	Novel gene
UBL5	-0.063	0.021	Reported gene
HYAL2	-0.064	0.021	Reported gene
DPP8	-0.064	0.021	Novel gene
XPO7	-0.064	0.021	Novel gene
TMED4	-0.064	0.021	Novel gene
RNMTL1	-0.064	0.021	Novel gene
TMEM200A	-0.064	0.021	Novel gene
LOC389348	-0.064	0.022	Novel gene
HBA1	-0.064	0.022	Novel gene
RAB9A	-0.064	0.022	Novel gene
ZBTB38	-0.064	0.022	Novel gene
PIH1D1	-0.064	0.022	Novel gene
LOC157567	-0.065	0.022	Novel gene
FAM122A	-0.065	0.022	Novel gene
C2CD2	-0.065	0.022	Novel gene
LDLR	-0.065	0.022	Novel gene
UPRT	-0.065	0.023	Novel gene
GRINA	-0.065	0.023	Novel gene
MRP63	-0.065	0.023	Novel gene

U2AF1L4	-0.065	0.023	Novel gene
PBXIP1	-0.065	0.023	Novel gene
TERF2	-0.065	0.023	Novel gene
RNASEH1P1	-0.065	0.023	Novel gene
TCERG1L	-0.065	0.023	Novel gene
TEX264	-0.065	0.023	Novel gene
GPR22	-0.065	0.024	Novel gene
BRAP	-0.066	0.024	Novel gene
SYNJ2BP	-0.066	0.024	Novel gene
PLEC	-0.066	0.024	Novel gene
CD37	-0.066	0.024	Novel gene
ZFC3H1	-0.066	0.024	Novel gene
FAM222B	-0.066	0.024	Novel gene
15-Sep	-0.066	0.024	Novel gene
LINC00526	-0.066	0.024	Novel gene
BRIX1	-0.066	0.025	Novel gene
FLJ20373	-0.066	0.025	Novel gene
ZNF524	-0.066	0.025	Novel gene
RRP8	-0.066	0.025	Novel gene
PIAS3	-0.066	0.025	Novel gene
MAP4K2	-0.066	0.025	Novel gene
RTCA	-0.066	0.025	Novel gene
MPZL2	-0.066	0.025	Reported gene
PPM1G	-0.067	0.025	Novel gene
DERA	-0.067	0.025	Novel gene
KLHL28	-0.067	0.026	Novel gene
SERINC2	-0.067	0.026	Novel gene
PTGES3	-0.067	0.026	Novel gene
DHRS4L2	-0.067	0.026	Novel gene
YWHAE	-0.067	0.026	Novel gene
IGF1R	-0.067	0.026	Novel gene
ZNF18	-0.067	0.026	Novel gene
AGPAT4-IT1	-0.067	0.026	Novel gene
TBC1D14	-0.067	0.027	Novel gene
TBRG1	-0.067	0.027	Novel gene
STX12	-0.067	0.027	Novel gene
BMP1	-0.067	0.027	Novel gene
NLE1	-0.068	0.027	Novel gene
PPP1R3D	-0.068	0.027	Novel gene
TP53BP1	-0.068	0.027	Novel gene
PDK2	-0.068	0.027	Novel gene
TBL3	-0.068	0.027	Novel gene
SLC27A5	-0.068	0.028	Novel gene
ICT1	-0.068	0.028	Novel gene
MPST	-0.068	0.028	Novel gene
CHEK2	-0.068	0.028	Novel gene
DCBLD2	-0.068	0.028	Novel gene
CUL4B	-0.068	0.028	Novel gene
RBBP9	-0.068	0.028	Novel gene
WIPF2	-0.068	0.028	Novel gene
NIPA2	-0.068	0.028	Novel gene
SYT11	-0.068	0.029	Novel gene
GOLPH3	-0.069	0.029	Novel gene

GGCX	-0.069	0.029	Novel gene
NARG2	-0.069	0.029	Novel gene
ELP4	-0.069	0.029	Novel gene
LOC91942	-0.069	0.029	Novel gene
PRPF4	-0.069	0.029	Novel gene
LOC399865	-0.069	0.029	Novel gene
SDHA	-0.069	0.029	Novel gene
GALNTL4	-0.069	0.030	Novel gene
SOX13	-0.069	0.030	Novel gene
TRIM56	-0.069	0.030	Novel gene
MYL5	-0.069	0.030	Novel gene
SRSF2	-0.069	0.030	Novel gene
SLAIN1	-0.069	0.030	Novel gene
RHOG	-0.069	0.030	Novel gene
RAI14	-0.069	0.030	Reported gene
CTSO	-0.070	0.030	Novel gene
MRPS11	-0.070	0.030	Novel gene
MESDC1	-0.070	0.031	Novel gene
FABP7	-0.070	0.031	Novel gene
ATP5B	-0.070	0.031	Novel gene
FAM129A	-0.070	0.031	Novel gene
LOC130617	-0.070	0.031	Novel gene
LRRN1	-0.070	0.031	Novel gene
BMI1	-0.070	0.031	Novel gene
KIAA1033	-0.070	0.031	Novel gene
ETHE1	-0.070	0.031	Novel gene
C6orf130	-0.070	0.032	Novel gene
ATP9B	-0.070	0.032	Novel gene
C10orf137	-0.070	0.032	Novel gene
SLC35D2	-0.070	0.032	Novel gene
SOD1	-0.070	0.032	Novel gene
PCID2	-0.070	0.032	Novel gene
ZNF510	-0.070	0.032	Novel gene
PRUNE	-0.070	0.032	Novel gene
NUP88	-0.071	0.033	Novel gene
DAAM2	-0.071	0.033	Novel gene
C4orf27	-0.071	0.033	Novel gene
FGFR3	-0.071	0.033	Novel gene
WDR77	-0.071	0.033	Novel gene
HAUS2	-0.071	0.033	Novel gene
HLA-DPB1	-0.071	0.033	Novel gene
DDX52	-0.071	0.033	Novel gene
SCFD1	-0.071	0.033	Reported gene
HS3ST6	-0.071	0.034	Novel gene
CKS1B	-0.071	0.034	Novel gene
GEMIN4	-0.071	0.034	Novel gene
FOXJ3	-0.071	0.034	Novel gene
STAB1	-0.071	0.034	Novel gene
SECISBP2L	-0.071	0.034	Novel gene
CYBRD1	-0.071	0.034	Novel gene
TOMM40L	-0.071	0.034	Novel gene
AP3M2	-0.071	0.034	Novel gene
LSM4	-0.071	0.035	Novel gene

CCND1	-0.071	0.035	Novel gene
METTL1	-0.071	0.035	Novel gene
APC	-0.072	0.035	Novel gene
PPP1R14C	-0.072	0.035	Novel gene
CNNM2	-0.072	0.035	Novel gene
LARP1	-0.072	0.035	Novel gene
UQCRH	-0.072	0.035	Novel gene
DHX38	-0.072	0.035	Novel gene
RNF43	-0.072	0.036	Novel gene
CD99L2	-0.073	0.036	Novel gene
SLC12A9	-0.073	0.036	Novel gene
FXYD5	-0.073	0.036	Novel gene
BAZ1B	-0.073	0.036	Novel gene
SLC48A1	-0.073	0.036	Novel gene
THAP6	-0.073	0.036	Novel gene
OXCT1	-0.073	0.036	Novel gene
YTHDF2P1	-0.073	0.036	Novel gene
LASP1	-0.073	0.037	Novel gene
PIAS4	-0.073	0.037	Novel gene
NLK	-0.073	0.037	Novel gene
MGST2	-0.073	0.037	Novel gene
DAB2	-0.074	0.037	Novel gene
MRPL39	-0.074	0.037	Novel gene
UBTD1	-0.074	0.037	Novel gene
TPPP	-0.074	0.037	Novel gene
DFFB	-0.074	0.037	Novel gene
ATP2C1	-0.074	0.038	Novel gene
DHX8	-0.074	0.038	Novel gene
VPS26B	-0.074	0.038	Novel gene
BCL2L13	-0.074	0.038	Novel gene
CRY1	-0.074	0.038	Novel gene
PDS5A	-0.074	0.038	Novel gene
ACADM	-0.074	0.038	Reported gene
PHB	-0.074	0.038	Novel gene
ATPAF2	-0.075	0.038	Novel gene
DYNC1I2	-0.075	0.039	Novel gene
CCDC51	-0.075	0.039	Novel gene
FSTL3	-0.075	0.039	Novel gene
H105E3	-0.075	0.039	Novel gene
SDHD	-0.075	0.039	Novel gene
FLJ32205	-0.075	0.039	Novel gene
TAF6L	-0.075	0.039	Novel gene
ATAD2	-0.075	0.039	Novel gene
RAB18	-0.075	0.039	Novel gene
CTSH	-0.075	0.040	Novel gene
G2E3	-0.075	0.040	Novel gene
CTC1	-0.075	0.040	Reported gene
LOC401033	-0.075	0.040	Novel gene
MAPK11	-0.075	0.040	Novel gene
NDUFB4	-0.075	0.040	Novel gene
HSPBP1	-0.075	0.040	Novel gene
ERRFI1	-0.076	0.040	Novel gene
NAGA	-0.076	0.040	Novel gene

PIP5K1A	-0.076	0.041	Novel gene
TEX29	-0.076	0.041	Novel gene
ZKSCAN5	-0.076	0.041	Novel gene
HYI	-0.076	0.041	Novel gene
R3HDM2	-0.076	0.041	Novel gene
ST3GAL5	-0.076	0.041	Novel gene
CCND2	-0.076	0.041	Novel gene
ANK2	-0.076	0.041	Novel gene
PHF17	-0.076	0.041	Novel gene
ABCC13	-0.076	0.042	Novel gene
PTPRN2	-0.077	0.042	Novel gene
CIRBP	-0.077	0.042	Novel gene
TMEM19	-0.077	0.042	Novel gene
LOC401505	-0.077	0.042	Novel gene
TCEB3	-0.077	0.042	Novel gene
CTCF	-0.077	0.042	Novel gene
PEG10	-0.077	0.042	Novel gene
TCEB1	-0.077	0.042	Novel gene
MAP6D1	-0.077	0.043	Novel gene
WFDC2	-0.077	0.043	Novel gene
UQCRC2	-0.077	0.043	Novel gene
SOCS2	-0.077	0.043	Novel gene
IDUA	-0.077	0.043	Novel gene
LOC51693	-0.077	0.043	Novel gene
SERPINA3	-0.077	0.043	Novel gene
HSPA5	-0.077	0.043	Novel gene
ANKRD24	-0.077	0.043	Novel gene
ZMYM1	-0.078	0.044	Novel gene
CIB2	-0.078	0.044	Novel gene
PRO1843	-0.078	0.044	Novel gene
RHOA	-0.078	0.044	Reported gene
ANKS1A	-0.078	0.044	Novel gene
MMP28	-0.078	0.044	Novel gene
RPS23	-0.078	0.044	Novel gene
LOC389792	-0.079	0.044	Novel gene
ATP6V1E1	-0.079	0.044	Novel gene
LOC51145	-0.079	0.045	Novel gene
FCGR3A	-0.079	0.045	Novel gene
LRRCC1	-0.079	0.045	Novel gene
FAM13B	-0.079	0.045	Novel gene
PDGFA	-0.079	0.045	Novel gene
RNF13	-0.079	0.045	Novel gene
FAM174B	-0.079	0.045	Reported gene
GCA	-0.080	0.045	Novel gene
TK2	-0.080	0.045	Novel gene
ANXA11	-0.080	0.045	Novel gene
GTF3C6	-0.080	0.046	Novel gene
LOC148203	-0.080	0.046	Novel gene
RABEPK	-0.080	0.046	Novel gene
TSPAN6	-0.080	0.046	Novel gene
LOC126208	-0.080	0.046	Novel gene
HEBP2	-0.080	0.046	Novel gene
TMEM140	-0.081	0.046	Novel gene

CCDC12	-0.081	0.046	Novel gene
MAN2A1	-0.081	0.047	Novel gene
RAD9A	-0.081	0.047	Novel gene
ZDHHC14	-0.081	0.047	Novel gene
LOC399979	-0.081	0.047	Novel gene
VWA5B2	-0.081	0.047	Novel gene
VCAM1	-0.081	0.047	Novel gene
SEC61G	-0.081	0.047	Novel gene
ELOVL1	-0.081	0.047	Novel gene
FLJ23529	-0.081	0.047	Novel gene
HDDC3	-0.082	0.048	Novel gene
ZFHX2	-0.082	0.048	Novel gene
FNBP1L	-0.082	0.048	Novel gene
ENY2	-0.082	0.048	Reported gene
SF3A3	-0.082	0.048	Novel gene
TRIB3	-0.082	0.048	Novel gene
UBA7	-0.082	0.048	Reported gene
COG3	-0.082	0.048	Novel gene
UBE2F	-0.082	0.048	Novel gene
EYA2	-0.083	0.049	Novel gene
SNTA1	-0.083	0.049	Novel gene
HNRNPU	-0.083	0.049	Novel gene
LOC114984	-0.083	0.049	Novel gene
CPNE2	-0.083	0.049	Novel gene
LOC388341	-0.083	0.049	Novel gene
OGFOD2	-0.083	0.049	Novel gene
C8orf42	-0.083	0.049	Novel gene
LOC400586	-0.083	0.049	Novel gene
NEO1	-0.083	0.050	Novel gene
ZNF436	-0.083	0.050	Novel gene
REEP6	-0.083	0.050	Novel gene
FRG1	-0.083	0.050	Novel gene

Supplemental Table S2. Significant pathways enriched by insomnia-related genes identified from *Sherlock* integrative analysis

Pathway ID	Pathway Terms	Associated genes proportion	P value	Corrected P value
KEGG:05016	Huntington's disease	0.073	5.58E-05	0.014
KEGG:05010	Alzheimer's disease	0.076	6.34E-05	0.015
KEGG:00190	Oxidative phosphorylation	0.083	1.09E-04	0.026
KEGG:03040	Spliceosome	0.082	1.17E-04	0.028
KEGG:05012	Parkinson's disease	0.077	1.95E-04	0.045
KEGG:04310	Wnt signaling pathway	0.077	2.07E-04	0.048

Supplemental Table S3. Significant GO-terms enriched by insomnia-related genes identified from *Sherlock* integrative analysis

GO ID	GO Terms	Associated genes proportion	P value	Corrected P value
GO:0003730	mRNA 3'-UTR binding	0.12	7.39E-05	0.018
GO:0003729	mRNA binding	0.069	1.01E-04	0.024
GO:0030529	Intracellular ribonucleoprotein complex	0.045	1.10E-05	0.003
GO:0044429	Mitochondrial part	0.041	3.22E-05	0.008
GO:0098798	Mitochondrial protein complex	0.082	6.08E-05	0.015
GO:0044455	Mitochondrial membrane part	0.069	1.01E-04	0.024
GO:0098803	Respiratory chain complex	0.11	1.82E-04	0.042
GO:0006275	Regulation of DNA replication	0.085	8.28E-05	0.020
GO:0006611	Protein export from nucleus	0.069	1.65E-04	0.039
GO:0051169	Nuclear transport	0.048	1.82E-04	0.042

Supplemental Table S4. Phenotype-related gene sets in WebGeslat resource significantly enriched by insomnia-associated genes identified from *Sherlock* integrative analysis

Gene Set	Description	Size	Expect	Ratio	P Value
HP:0002060	Abnormality of the cerebrum	1313	29.10	1.62	4.88E-05
HP:0100547	Abnormality of forebrain morphology	1334	29.56	1.59	7.66E-05
HP:0002011	Morphological abnormality of the central nervous system	1909	42.30	1.37	4.17E-04
HP:0012758	Neurodevelopmental delay	1474	32.66	1.47	5.19E-04
HP:0011804	Abnormality of muscle physiology	1758	38.96	1.39	7.28E-04
HP:0012443	Abnormality of brain morphology	1741	38.58	1.37	1.16E-03
HP:0008972	Decreased activity of mitochondrial respiratory chain	55	1.22	4.92	1.21E-03
HP:0001252	Muscular hypotonia	1187	26.30	1.52	1.23E-03
HP:0011922	Abnormal activity of mitochondrial respiratory chain	56	1.24	4.84	1.33E-03
HP:0001263	Global developmental delay	1238	27.43	1.49	1.47E-03
HP:0003110	Abnormality of urine homeostasis	455	10.08	1.98	1.74E-03
HP:0003808	Abnormal muscle tone	1467	32.51	1.42	2.00E-03
HP:0002536	Abnormal cortical gyration	211	4.68	2.57	2.09E-03
HP:0002269	Abnormality of neuronal migration	274	6.07	2.31	2.44E-03
HP:0012759	Neurodevelopmental abnormality	1975	43.76	1.30	2.55E-03
HP:0001640	Cardiomegaly	65	1.44	4.17	2.88E-03
HP:0001250	Seizures	1321	29.27	1.43	2.94E-03
HP:0002977	Aplasia/Hypoplasia involving the central nervous system	1157	25.64	1.48	3.01E-03
HP:0100833	Neoplasm of the small intestine	14	0.31	9.67	3.21E-03
HP:0001166	Arachnodactyly	89	1.97	3.55	3.25E-03
HP:0002538	Abnormality of the cerebral cortex	226	5.01	2.40	3.72E-03
HP:0003287	Abnormality of mitochondrial metabolism	116	2.57	3.11	3.79E-03
HP:0009125	Lipodystrophy	93	2.06	3.40	4.16E-03
HP:0000298	Mask-like facies	49	1.09	4.60	4.21E-03
HP:0001098	Abnormal fundus morphology	939	20.81	1.54	4.42E-03
HP:0002416	Subependymal cysts	5	0.11	18.05	4.65E-03
HP:0009659	Partial absence of thumb	5	0.11	18.05	4.65E-03
HP:0004329	Abnormal morphology of the posterior segment of the globe	942	20.87	1.53	4.66E-03
HP:0008316	Abnormal mitochondria in muscle tissue	32	0.71	5.64	5.03E-03
HP:0010993	Abnormality of the cerebral subcortex	643	14.25	1.68	5.21E-03
HP:0001290	Generalized hypotonia	718	15.91	1.63	5.28E-03
HP:0003355	Aminoaciduria	123	2.73	2.94	5.42E-03
HP:0002938	Lumbar hyperlordosis	52	1.15	4.34	5.45E-03
HP:0000079	Abnormality of the urinary system	1321	29.27	1.40	5.64E-03
HP:0002352	Leukoencephalopathy	124	2.75	2.91	5.69E-03
HP:0001238	Slender finger	100	2.22	3.16	6.21E-03
HP:0007364	Aplasia/Hypoplasia of the cerebrum	961	21.30	1.50	6.42E-03
HP:0003546	Exercise intolerance	77	1.71	3.52	6.69E-03
HP:0001920	Renal artery stenosis	18	0.40	7.52	6.74E-03

HP:0008776	Abnormal renal artery morphology	18	0.40	7.52	6.74E-03
HP:0001014	Angiokeratoma	6	0.13	15.04	6.87E-03
HP:0012103	Abnormality of the mitochondrion	128	2.84	2.82	6.88E-03
HP:0003367	Abnormality of the femoral neck	102	2.26	3.10	6.91E-03
HP:0031650	Abnormal atrioventricular valve physiology	102	2.26	3.10	6.91E-03
HP:0100631	Neoplasm of the adrenal gland	35	0.78	5.16	6.97E-03
HP:0011276	Vascular skin abnormality	409	9.06	1.88	7.22E-03
HP:0002376	Developmental regression	216	4.79	2.30	7.48E-03
HP:0200037	Skin vesicle	19	0.42	7.13	7.88E-03
HP:0002673	Coxa valga	57	1.26	3.96	8.05E-03
HP:0000980	Pallor	105	2.33	3.01	8.07E-03
HP:0001347	Hyperreflexia	556	12.32	1.70	8.24E-03
HP:0009124	Abnormal adipose tissue morphology	160	3.55	2.54	8.28E-03
HP:0000544	External ophthalmoplegia	37	0.82	4.88	8.50E-03
HP:0002808	Kyphosis	315	6.98	2.01	8.52E-03
HP:0001257	Spasticity	559	12.39	1.70	8.76E-03
HP:0007367	Atrophy/Degeneration affecting the central nervous system	523	11.59	1.73	8.78E-03
HP:0002815	Abnormality of the knee	253	5.61	2.14	9.10E-03
HP:0003207	Arterial calcification	20	0.44	6.77	9.12E-03
HP:0001373	Joint dislocation	285	6.32	2.06	9.13E-03
HP:0012372	Abnormal eye morphology	1747	38.71	1.29	9.17E-03
HP:0001582	Redundant skin	59	1.31	3.82	9.30E-03
HP:0002577	Abnormality of the stomach	352	7.80	1.92	9.42E-03
HP:0006980	Progressive leukoencephalopathy	7	0.16	12.89	9.48E-03
HP:0000587	Abnormality of the optic nerve	600	13.30	1.65	9.55E-03
HP:0012072	Aciduria	164	3.63	2.48	9.68E-03
HP:0011121	Abnormality of skin morphology	1447	32.06	1.34	1.01E-02
HP:0031605	Abnormality of fundus pigmentation	21	0.47	6.45	1.05E-02
HP:0100834	Neoplasm of the large intestine	61	1.35	3.70	1.07E-02
HP:0000670	Carious teeth	139	3.08	2.60	1.11E-02
HP:0000240	Abnormality of skull size	996	22.07	1.45	1.12E-02
HP:0001339	Lissencephaly	114	2.53	2.77	1.24E-02
HP:0040223	Pulmonary hemorrhage	8	0.18	11.28	1.25E-02
HP:0000951	Abnormality of the skin	1551	34.37	1.31	1.27E-02
HP:0001939	Abnormality of metabolism/homeostasis	1819	40.31	1.27	1.30E-02
HP:0002974	Radioulnar synostosis	63	1.40	3.58	1.22E-02
HP:0011277	Abnormality of the urinary system physiology	730	16.18	1.55	1.30E-02
HP:0100749	Chest pain	64	1.42	3.53	1.30E-02
HP:0004934	Vascular calcification	23	0.51	5.89	1.35E-02
HP:0000252	Microcephaly	771	17.09	1.52	1.35E-02
HP:0001276	Hypertonia	696	15.42	1.56	1.40E-02
HP:0040195	Decreased head circumference	773	17.13	1.52	1.40E-02
HP:0003072	Hypercalcemia	43	0.95	4.20	1.44E-02
HP:0100539	Periorbital edema	43	0.95	4.20	1.44E-02
HP:0001681	Angina pectoris	25	0.55	5.42	1.70E-02
HP:0002036	Hiatus hernia	24	0.53	5.64	1.52E-02

HP:0001279	Syncope	44	0.98	4.10	1.55E-02
HP:0001836	Camptodactyly of toe	9	0.20	10.03	1.58E-02
HP:0025031	Abnormality of the digestive system	1926	42.68	1.24	1.58E-02
HP:0008046	Abnormality of the retinal vasculature	178	3.94	2.28	1.60E-02
HP:0000969	Edema	340	7.53	1.86	1.61E-02
HP:0003366	Abnormality of the femoral neck or head region	121	2.68	2.61	1.69E-02
HP:0010876	Abnormality of circulating protein level	338	7.49	1.87	1.53E-02
HP:0011915	Cardiovascular calcification	25	0.55	5.42	1.70E-02
HP:0100634	Neuroendocrine neoplasm	25	0.55	5.42	1.70E-02
HP:0200034	Papule	69	1.53	3.27	1.76E-02
HP:0003712	Skeletal muscle hypertrophy	151	3.35	2.39	1.77E-02
HP:0002167	Neurological speech impairment	789	17.48	1.49	1.81E-02
HP:0000282	Facial edema	46	1.02	3.92	1.81E-02
HP:0001138	Optic neuropathy	46	1.02	3.92	1.81E-02
HP:0000602	Ophthalmoplegia	213	4.72	2.12	1.82E-02

Supplemental Table S5. Drug-related gene sets in GLAD4U resource significantly enriched by insomnia-associated genes identified from *Sherlock* integrative analysis

Gene Set	Description	Size	Expect	Ratio	P Value
PA162364313	bezafibrate	20	0.40	9.91	6.09E-04
PA450536	molybdenum	24	0.48	8.26	1.25E-03
PA449942	hydroxyurea	86	1.74	4.03	1.74E-03
PA451673	l-threonine	848	17.12	1.75	1.90E-03
PA164712853	Iron Preparations	212	4.28	2.57	3.88E-03
PA450087	iron	212	4.28	2.57	3.88E-03
PA450744	oxygen	318	6.42	2.18	5.25E-03
PA449789	glycine	223	4.50	2.44	5.65E-03
PA164776929	lipoic acid	59	1.19	4.20	6.65E-03
PA448784	carbachol	61	1.23	4.06	7.64E-03
PA164712614	Colchicine derivatives	7	0.14	14.15	7.97E-03
PA164713367	Tumour Detection	206	4.16	2.40	9.08E-03
PA451704	tobramycin	116	2.34	2.99	9.10E-03
PA164746343	sulodexide	8	0.16	12.39	1.05E-02
PA164713204	Protein kinase inhibitors	490	9.89	1.82	1.06E-02
PA451565	sulindac	23	0.46	6.46	1.07E-02
PA452612	vinyl chloride	23	0.46	6.46	1.07E-02
PA166122986	radiotherapy	121	2.44	2.87	1.13E-02
PA449917	hydrogen peroxide	152	3.07	2.61	1.20E-02
PA164712734	Enzymes	1996	40.29	1.34	1.32E-02
PA164712467	Antiinflammatory/antirheumatic agents in combination with corticosteroids	9	0.18	11.01	1.33E-02
PA164745443	dydrogesterone	9	0.18	11.01	1.33E-02
PA10364	memantine	25	0.50	5.94	1.35E-02
PA164712693	Diphtheria vaccines	25	0.50	5.94	1.35E-02
PA451644	thalidomide	46	0.93	4.31	1.36E-02
PA166163262	sodium ascorbate	46	0.93	4.31	1.36E-02
PA164712750	Ethers	99	2.00	3.00	1.50E-02
PA164752658	porfimer	10	0.20	9.91	1.64E-02
PA164712314	Actinomycines	193	3.90	2.31	1.65E-02
PA151917012	dactinomycin	193	3.90	2.31	1.65E-02
PA450954	phosphorus	51	1.03	3.89	1.93E-02
PA153590860	camptothecin	105	2.12	2.83	1.95E-02
PA166131608	dovitinib	11	0.22	9.01	1.98E-02
PA450644	nitroglycerin	11	0.22	9.01	1.98E-02
PA1102	qt-prolonging drugs	29	0.59	5.12	2.02E-02
PA151958596	curcumin	53	1.07	3.74	2.19E-02
PA164712582	calcium	811	16.37	1.53	2.28E-02
PA134687907	formoterol	12	0.24	8.26	2.34E-02
PA10816	ezetimibe	12	0.24	8.26	2.34E-02
PA451898	vitamin c	55	1.11	3.60	2.48E-02
PA449776	l-glutamic acid	174	3.51	2.28	2.49E-02
PA449376	disulfiram	13	0.26	7.62	2.73E-02

PA165946121	fasudil	13	0.26	7.62	2.73E-02
PA449563	exemestane	13	0.26	7.62	2.73E-02
PA450280	l-lysine	874	17.64	1.47	3.01E-02
PA452611	epipodophyllotoxin	117	2.36	2.54	3.12E-02
PA450993	podofilox	117	2.36	2.54	3.12E-02
PA164754912	fluocinolone acetonide	14	0.28	7.08	3.15E-02
PA451843	l-valine	88	1.78	2.81	3.26E-02
PA164754913	netilmicin	90	1.82	2.75	3.54E-02
PA164712466	Antiinflammatory/antirheumatic Agents In Combination	36	0.73	4.13	3.56E-02
PA164713246	Salt solutions	36	0.73	4.13	3.56E-02
PA450196	letrozole	15	0.30	6.61	3.58E-02
PA450203	leuprolide	15	0.30	6.61	3.58E-02
PA164713003	Other alkylating agents	62	1.25	3.20	3.64E-02
PA449021	citric acid	92	1.86	2.69	3.84E-02
PA166114906	bosutinib	16	0.32	6.19	4.04E-02
PA452620	tegafur	16	0.32	6.19	4.04E-02
PA164712585	Calcium Homeostasis	193	3.90	2.05	4.22E-02
PA166160055	cholic acid	39	0.79	3.81	4.36E-02
PA165860812	berberine	17	0.34	5.83	4.52E-02
PA448873	cellulose	40	0.81	3.72	4.64E-02
PA154081778	4-methylumbelliferone	41	0.83	3.62	4.94E-02
PA451261	rituximab	41	0.83	3.62	4.94E-02
PA10489	selenium supplements	18	0.36	5.50	5.00E-02
PA164712917	Muscle Relaxants	18	0.36	5.50	5.00E-02

Supplemental Table S6. Drug-related gene sets in DrugBank resource significantly enriched by insomnia-associated genes identified from *Sherlock* integrative analysis

Gene Set	Description	Size	Expect	Ratio	P Value
DB04141	2-Hexyloxy-6-Hydroxymethyl-Tetrahydro-Pyran-3,4,5-Triol	15	0.33	12.20	2.36E-04
DB09270	Ubidecarenone	6	0.13	15.26	6.66E-03
DB13257	Ferrous sulfate	20	0.44	6.86	8.70E-03
DB01119	Diazoxide	7	0.15	13.08	9.20E-03
DB07763	(5S)-3-ANILINO-5-(2,4-DIFLUOROPHENYL)-5-METHYL-1,3-OXAZOLIDINE-2,4-DIONE	10	0.22	9.15	1.89E-02
DB08330	METHYL (2Z)-3-METHOXY-2-{2-[(E)-2-PHENYLVINYL]PHENYL}ACRYLATE	10	0.22	9.15	1.89E-02
DB08453	2-NONYL-4-HYDROXYQUINOLINE N-OXIDE	10	0.22	9.15	1.89E-02
DB08690	UBIQUINONE-2	10	0.22	9.15	1.89E-02
DB04799	5-n-undecyl-6-hydroxy-4,7-dioxobenzothiazole	10	0.22	9.15	1.89E-02
DB07778	FAMOXADONE	10	0.22	9.15	1.89E-02
DB07401	METHYL (2Z)-2-(2-{[6-(2-CYANOPHENOXY)PYRIMIDIN-4-YL]OXY}PHENYL)-3-METHOXYACRYLATE	10	0.22	9.15	1.89E-02
DB00139	Succinic acid	27	0.59	5.09	2.00E-02
DB00132	Alpha-Linolenic Acid	11	0.24	8.32	2.28E-02
DB04224	Oleic Acid	12	0.26	7.63	2.69E-02
DB00159	Icosapent	13	0.28	7.04	3.14E-02
DB09462	Glycerin	15	0.33	6.10	4.11E-02
DB12267	Brigatinib	16	0.35	5.72	4.63E-02

Supplemental Table S7. ***Sherlock* integrative analysis identifies 184 genes as insomnia risk genes in validation samples (GTEx brain cortex samples)**

Gene	LBF	*Sherlock*-based P value	GWAS catalog
FAM86DP	-0.376	2.303E-04	Novel gene
AP1G2	-0.380	5.155E-04	Novel gene
C10orf111	-0.396	5.758E-04	Novel gene
BTG1	-0.404	9.378E-04	Novel gene
TAF8	-0.413	1.311E-03	Novel gene
TXN	-0.423	1.579E-03	Novel gene
CSAD	-0.426	1.727E-03	Novel gene
ENSG00000270021	-0.430	1.925E-03	Novel gene
ZBTB26	-0.431	2.468E-03	Novel gene
HSD17B7P2	-0.437	2.660E-03	Novel gene
ENSG00000227540	-0.441	2.863E-03	Novel gene
DIS3L	-0.443	3.016E-03	Novel gene
ENSG00000214198	-0.447	3.016E-03	Novel gene
MXRA8	-0.449	3.526E-03	Novel gene
SATB2-AS1	-0.451	3.718E-03	Novel gene
UQCRFS1	-0.454	3.987E-03	Novel gene
AMOTL1	-0.455	4.135E-03	Novel gene
MAPK14	-0.459	4.491E-03	Novel gene
VWA3B	-0.472	4.672E-03	Novel gene
GART	-0.478	4.996E-03	Novel gene
NPY5R	-0.480	5.473E-03	Novel gene
MYOZ3	-0.488	5.643E-03	Novel gene
URB1	-0.493	6.032E-03	Novel gene
RYR1	-0.494	6.038E-03	Novel gene
HLA-DMB	-0.497	6.367E-03	Novel gene
PSD2	-0.498	6.855E-03	Novel gene
ENSG00000226237	-0.498	7.074E-03	Novel gene
ENSG00000239415	-0.499	7.206E-03	Novel gene
ANKRD13B	-0.501	7.403E-03	Novel gene
SCNN1D	-0.507	7.880E-03	Novel gene
DNAJC12	-0.514	7.952E-03	Novel gene
SP110	-0.514	8.451E-03	Novel gene
GTSF1	-0.514	8.599E-03	Novel gene
LEFTY1	-0.518	8.900E-03	Novel gene
EXOC4	-0.521	9.235E-03	Novel gene
ZNF678	-0.522	0.010	Novel gene
MC1R	-0.523	0.010	Novel gene
ITGA11	-0.527	0.010	Novel gene
GPR98	-0.529	0.010	Novel gene
DHRS4	-0.536	0.011	Novel gene
C1orf194	-0.537	0.011	Novel gene
OR7E29P	-0.538	0.011	Novel gene
VWA8	-0.539	0.011	Novel gene
CHEK1	-0.540	0.012	Novel gene
MEGF8	-0.541	0.012	Novel gene
C6orf201	-0.542	0.012	Reported gene
TSC1	-0.546	0.012	Novel gene
STARD6	-0.546	0.013	Novel gene
C8orf31	-0.553	0.013	Novel gene

KCNJ16	-0.554	0.013	Novel gene
ENSG00000213839	-0.554	0.014	Novel gene
ENSG00000236915	-0.557	0.014	Novel gene
ENSG00000216921	-0.559	0.014	Novel gene
DHX36	-0.559	0.014	Novel gene
ENSG00000273075	-0.561	0.015	Novel gene
HEBP2	-0.563	0.015	Novel gene
CST3	-0.564	0.015	Novel gene
CYB5R3	-0.565	0.016	Novel gene
WDR36	-0.566	0.016	Novel gene
TIPIN	-0.567	0.016	Novel gene
ENSG00000272221	-0.567	0.016	Novel gene
RAB37	-0.568	0.016	Novel gene
ENSG00000253326	-0.568	0.017	Novel gene
DYDC2	-0.568	0.017	Novel gene
TCTN2	-0.570	0.017	Novel gene
FGL1	-0.572	0.017	Novel gene
LDHA	-0.572	0.018	Novel gene
ENSG00000236155	-0.573	0.018	Novel gene
BDKRB1	-0.573	0.018	Novel gene
RELL2	-0.574	0.019	Novel gene
CRYZ	-0.577	0.019	Novel gene
YARS	-0.578	0.019	Novel gene
TRAF3IP2-AS1	-0.582	0.019	Novel gene
PINLYP	-0.584	0.020	Novel gene
IL20RA	-0.584	0.020	Novel gene
UBE2G2	-0.584	0.020	Novel gene
TEX264	-0.585	0.020	Novel gene
ZC3HC1	-0.585	0.021	Novel gene
GCOM2	-0.587	0.021	Novel gene
ENSG00000271643	-0.588	0.021	Novel gene
ENSG00000222020	-0.589	0.021	Novel gene
LINC00115	-0.590	0.022	Novel gene
NMUR2	-0.592	0.022	Novel gene
DNAH1	-0.593	0.022	Novel gene
ENSG00000214135	-0.593	0.023	Novel gene
ENSG00000262528	-0.594	0.023	Novel gene
FGFR3	-0.594	0.023	Novel gene
LINC00562	-0.595	0.023	Novel gene
KIAA0391	-0.595	0.024	Novel gene
XRCC3	-0.596	0.024	Novel gene
ENSG00000232063	-0.596	0.024	Novel gene
SCN4B	-0.597	0.025	Novel gene
ENSG00000248673	-0.597	0.025	Novel gene
SIGLEC10	-0.598	0.025	Novel gene
CDCA5	-0.598	0.025	Novel gene
NKTR	-0.598	0.026	Novel gene
ENSG00000272505	-0.598	0.026	Novel gene
ENSG00000256594	-0.599	0.026	Novel gene
ACY3	-0.599	0.027	Novel gene
RUVBL2	-0.599	0.027	Novel gene
ENSG00000240685	-0.601	0.027	Novel gene
CAMK1G	-0.602	0.027	Novel gene

REV3L	-0.602	0.028	Novel gene
ENSG00000258959	-0.602	0.028	Novel gene
ENSG00000231105	-0.603	0.028	Novel gene
DUS2	-0.603	0.028	Novel gene
DALRD3	-0.605	0.029	Novel gene
LOH12CR1	-0.606	0.029	Novel gene
ENSG00000271868	-0.606	0.029	Novel gene
JRK	-0.606	0.030	Novel gene
TMEM219	-0.606	0.030	Novel gene
RECQL	-0.606	0.030	Novel gene
KIF7	-0.607	0.030	Novel gene
TOX4	-0.608	0.031	Novel gene
ZNF782	-0.608	0.031	Novel gene
WASF3	-0.609	0.031	Novel gene
TPBGL	-0.610	0.031	Novel gene
CDC42SE2	-0.610	0.032	Novel gene
ENSG00000257086	-0.610	0.032	Novel gene
7-Sep	-0.611	0.032	Novel gene
SKA2	-0.612	0.033	Novel gene
AK5	-0.612	0.033	Reported gene
GPR155	-0.612	0.033	Novel gene
ENDOG	-0.613	0.033	Novel gene
ALPK3	-0.613	0.034	Novel gene
FAM178B	-0.614	0.034	Novel gene
ARL9	-0.614	0.034	Novel gene
PFAS	-0.614	0.034	Novel gene
RSPO2	-0.614	0.035	Novel gene
ZNF764	-0.615	0.035	Novel gene
ENSG00000205037	-0.615	0.035	Novel gene
ANKRD20A19P	-0.615	0.035	Novel gene
DIEXF	-0.615	0.036	Novel gene
ENSG00000218227	-0.617	0.036	Novel gene
ENSG00000270607	-0.617	0.036	Novel gene
RHOF	-0.617	0.037	Novel gene
NUS1	-0.617	0.037	Novel gene
EIF6	-0.617	0.037	Novel gene
CSRP2	-0.617	0.038	Novel gene
ENSG00000259658	-0.618	0.038	Novel gene
ITGAV	-0.618	0.038	Novel gene
WIPF3	-0.618	0.038	Novel gene
GALNT2	-0.618	0.039	Novel gene
ENSG00000183562	-0.619	0.039	Novel gene
PARD3B	-0.619	0.039	Novel gene
ZFYVE20	-0.619	0.039	Novel gene
LINC00662	-0.619	0.040	Novel gene
GKN1	-0.620	0.040	Novel gene
P4HA2	-0.620	0.040	Novel gene
ENSG00000257433	-0.621	0.041	Novel gene
ENSG00000269473	-0.621	0.041	Novel gene
KHNYN	-0.621	0.041	Novel gene
SRGAP2C	-0.622	0.041	Novel gene
CCDC144B	-0.622	0.041	Novel gene
ENSG00000224376	-0.623	0.042	Novel gene

ENSG00000235448	-0.624	0.042	Novel gene
HCG17	-0.624	0.042	Novel gene
ENSG00000229407	-0.625	0.043	Novel gene
ZNF253	-0.625	0.043	Novel gene
CCDC183-AS1	-0.627	0.043	Novel gene
ENSG00000261037	-0.628	0.044	Novel gene
GDF6	-0.628	0.044	Novel gene
CHRAC1	-0.630	0.044	Novel gene
LEPR	-0.630	0.044	Novel gene
VN1R108P	-0.630	0.045	Novel gene
ENSG00000259251	-0.630	0.045	Novel gene
FOLH1	-0.630	0.045	Novel gene
VAMP1	-0.631	0.045	Novel gene
SMYD3	-0.631	0.046	Novel gene
FER1L4	-0.631	0.046	Novel gene
DNAL1	-0.633	0.046	Novel gene
ROM1	-0.633	0.047	Novel gene
CDKN2B	-0.633	0.047	Novel gene
LGALS3	-0.633	0.047	Novel gene
A4GNT	-0.634	0.047	Novel gene
ENSG00000265519	-0.634	0.048	Novel gene
ENSG00000258985	-0.635	0.048	Novel gene
SWAP70	-0.635	0.048	Novel gene
FAM69A	-0.635	0.048	Novel gene
ZNF136	-0.635	0.049	Novel gene
PSD3	-0.636	0.049	Novel gene
NELFCD	-0.636	0.049	Novel gene
PREX2	-0.637	0.049	Novel gene
EPB41L5	-0.637	0.050	Novel gene

Supplemental Table S8. Five Insomnia-associated risk genes identified by Sherlock integrative analysis between two eQTL datasets

Gene name	Discovery stage				Validation stage				*Differential expression in Brain between insomnia and control*
	Supporting SNP [a]	*eSNP-based* **Pvalue** [b]	*GWAS-based* **P value** [c]	*Sherlock-* **based P value** [d]	*Supporting SNP* [a]	*eSNP-based* **Pvalue** [b]	*GWAS-based* **P value** [c]	*Sherlock-* **based P value** [d]	
LDHA	rs8131143	9.73E-06	8.97E-01	9.11E-03	rs10741758	7.98E-06	7.73E-01	1.77E-02	4.40E-02
DALRD3	rs2782892	8.43E-06	3.71E-01	1.13E-02	rs6795772	9.70E-06	2.34E-02	2.85E-02	5.00E-05
TEX264	rs6762880	8.57E-06	4.35E-01	3.29E-02	rs6778196	8.77E-06	6.09E-01	2.04E-02	3.89E-01
FGFR3	rs1407228	9.06E-06	1.14E-01	2.35E-02	rs2234909	8.08E-06	6.58E-01	2.31E-02	7.75E-01
HEBP2	rs766310	6.26E-06	6.25E-01	4.62E-02	rs4896353	7.36E-06	8.75E-01	1.51E-02	3.20E-02

Note: [a] SNP influences the expression level of risk gene.

[b] P-value from expression quantitative trait analysis of Myers et al.

[c] P-value from GWAS on insomnia of Jansen et al.

[d] P–value from calculation based on the*Sherlock* Bayesianintegrative analysis. In light of*Sherlock* tool uses finite times of permutation test to calculate the Pvalue for each gene, some of these top-ranked genes have distinct logarithm of the Bayes factor values but obtain the same rankings (namely, their P values are same).

Supplemental Table S9. The co-expression analysis of the 5 identified genes based on the Pearson correlation method in all samples of GSE40562

	LDHA	*DALRD3*	*TEX264*	*FGFR3*	*HEBP2*
LDHA	1.00	0.62	-0.40	-0.34	0.32
DALRD3	0.62	1.00	-0.48	-0.09	0.54
TEX264	-0.40	-0.48	1.00	0.55	-0.31
FGFR3	-0.34	-0.09	0.55	1.00	0.19
HEBP2	0.32	0.54	-0.31	0.19	1.00

Supplemental Table S10. The co-expression analysis of the 5 identified genes based on the Pearson correlation method in insomnia patients of GSE40562

	LDHA	*DALRD3*	*TEX264*	*FGFR3*	*HEBP2*
LDHA	1.00	0.06	-0.18	-0.34	-0.17
DALRD3	0.06	1.00	-0.31	0.21	-0.24
TEX264	-0.18	-0.31	1.00	0.58	-0.12
FGFR3	-0.34	0.21	0.58	1.00	0.14
HEBP2	-0.17	-0.24	-0.12	0.14	1.00
